# Predictive value of CHADS_2_ and CHA_2_DS_2_-VASc scores for coronary artery lesions and in-hospital prognosis of patients with acute ST-segment elevation myocardial infarction

**DOI:** 10.1186/s12872-021-02257-2

**Published:** 2021-09-15

**Authors:** Xiaoli Li, Zhen Zeng, Xinchun Yang, Hongjiang Wang

**Affiliations:** 1 Department of Cardiology, Beijing Chaoyang Hospital, Capital Medical University; Heart Center and Beijing Key Laboratory of Hypertension, Beijing Chaoyang Hospital, Capital Medical University, No.8 Gongti South Road, Chaoyang District, Beijing, 100020 China; 2 Geriatric Department, Chui Yang Liu Hospital, Tsinghua University, Beijing, 100022 China

**Keywords:** Acute ST-segment elevation myocardial infarction, CHADS_2_ score, CHA_2_DS_2_-VASc score, Gensini score, Adverse events during hospitalization

## Abstract

**Objective:**

To evaluate the predictive value of CHADS_2_ and CHA_2_DS_2_-VASc scores for coronary artery lesions and in-hospital prognosis of patients with acute ST-segment elevation myocardial infarction (STEMI).

**Methods:**

A total of 524 patients who were diagnosed with STEMI from January 2016 to August 2017 were retrospectively reviewed. The correlation between CHADS_2_ and CHA_2_DS_2_-VASc scores with the patients’ clinical data, number of coronary lesions, Gensini scores, the target vessel and hospitalization time and in-hospital adverse events (AEs) was analyzed.

**Results:**

The number of coronary lesions in STEMI patients was mainly single and double lesions. The CHADS_2_ and CHA_2_DS_2_-VASc scores were not meaningful for predicting the number of coronary lesions. However, for left main coronary artery lesion, CHADS_2_ score was significantly increased when the number increased (*P* < 0.05), but CHA_2_DS_2_-VASc score showed no statistical difference (*P* > 0.05). The incidence of target lesions in STMEI patients was mainly left anterior descending coronary artery (LAD) and right coronary artery (RCA). The two scores were not meaningful for predicting target lesions (*P* > 0.05). For the severity of coronary lesions, there was positive correlation between CHADS_2_ score with Gensini score (*P* < 0.05), but no exact correlation between CHA_2_DS_2_-VASc score and Gensini score (*P* > 0.05). The stratifications of CHADS_2_ score and CHA_2_DS_2_-VASc score were significantly associated with hospitalization time and adverse events during hospitalization (*P* < 0.05). The high score group had longer hospitalization time and more AEs during hospitalization than the low score group and the middle group statistically (*P* < 0.05).

**Conclusion:**

CHADS_2_ score had a certain value to predict the severity of coronary lesion and the presence of left main coronary artery in STEMI. The CHA_2_DS_2_-VASc score had no predictive ability to do it. There was no significant value in predicting the number of coronary lesions and the location of the target lesions in STEMI patients. However, both scores had the predictive ability for patient hospitalization and AEs during hospitalization.

## Introduction

Coronary atherosclerotic heart disease (CAHD) is one of the most serious diseases threatening human health. In recent years, the incidence rate and mortality of CAHD have increased significantly [1]. Acute myocardial infarction (AMI), especially acute ST-segment elevated myocardial infarction (STEMI), is one of the most serious CAHDs, characterized by acute outlet, rapid progression and high mortality [2]. Many studies have reported that the occurrence and development of CAHD are significantly correlated with hypertension, diabetes, hyperlipidemia, long-term smoking, age, gender and other factors [3]. Besides, when patients with CAHD combined with multiple high-risk factors are confirmed as multivessel coronary artery disease by coronary angiography, the risk of AMI is higher and the condition can be more complex [4]. If the disease can be risk stratified in the early stage and high-risk patients can be identified, more effective interferences can be applied to reduce the incidence of AMI and improve the prognosis of patients. Currently, the commonly used scoring methods for AMI include Gensini and SYNTAX scoring system according to the shape of vascular lesions [5, 6]. Moreover, the TIMI and GRACE scoring system are widely used in evaluating the prognosis [7]. However, these scoring methods have different advantages and limitations in the treatment options and prognosis guidance of patients with AMI.

The superiority of CHADS_2_ and CHA_2_DS_2_-VASc scores in predicting the stroke risk of patients with nonvalvular atrial fibrillation have been proved [8, 9]. Although the two scales were first used in AMI patients without atrial fibrillation, investigators have focused on the utility of CHADS_2_ and CHA_2_DS_2_-VASc scores in predicting CAHD as many variables in the two scales are closely associated with the disease [10, 11]. They have reported that CHA_2_DS_2_-VASc score could be a potential independent predictor of acute stent thrombosis in STEMI patients treated with primary percutaneous coronary intervention [12]. Besides, CHA_2_DS_2_-VASc score is an applicable risk scoring system to assess the risk of development of portal vein thrombosis in patients with prosthetic valve [13]. However, few studies analyzed the potential value of CHADS_2_ and CHA_2_DS_2_-VASc scores in the prediction of ASTEMI. Besides, the predictive value of CHADS_2_ and CHA_2_DS_2_-VASc scores for in-hospital prognosis deserves to be explored.

This retrospective study reviewed the clinical data of patients with ASTEMI, including general patient data, history, vital signs, biochemical indicators, echocardiography results, coronary angiography, Gensini scores, CHADS_2_ and CHA_2_DS_2_-VASc scores. Based on these data, we aimed to evaluate the predictive value of CHADS_2_ and CHA_2_DS_2_-VASc scores for coronary artery lesions and in-hospital prognosis in patients with acute STEMI.

## Patients and methods

The eligibility criteria were as follows. The inclusion criteria included age above 18 years, STEMI rather than acute non-STEMI and consent to receipt of emergency coronary angiography. The exclusion criteria included disturbance of consciousness, blood pressure below 80/50 mmHg, heart rate below 40 beats/min, a history of hematological diseases or malignancy and a life expectancy less than 1 year, recent history of haemorrhage, being unable to tolerate aspirin or clopidogrel and other antiplatelet drugs, and acute cerebral apoplexy. According to the eligibility criteria, the records of 524 patients with a diagnosis of ASTEMI who received emergency coronary angioplasty from January 2016 to August 2017 at Beijing Chaoyang Hospital were reviewed.

The study protocol was approved by the Ethics Committee of Beijing Chaoyang Hospital and informed consent was obtained from all study subjects. All methods were carried out in accordance with relevant guidelines and regulations.

STEMI patients were given aspirin 300 mg and clopidogrel 600 mg before emergency coronary angiography. After coronary angiography and interventional therapy were performed, statins were given to each patient. According to the blood pressure, heart rate, cardiac function level, β receptor blockers, ACEI and diuretics were provided individually. Then, all subjects were evaluated by CHADS_2_ and CHA_2_DS_2_-VASc scores [8, 9]. According to the CHADS_2_ scores, patients were divided into the low group (a score of 0–1), the middle group (a score of 2–3) and the high group (a score of 4–6). Patients evaluated by CHA_2_DS_2_-VASc was divided into the low group (a score of 0–2 points), the middle group (a score of 3–4) and high group (a score of 5–9).

Clinical data were recorded, including age, gender, history (hypertension, hyperlipidemia, atrial fibrillation, diabetes, hyperuricemia, peripheral arteriosclerosis, cerebral apoplexy, transient ischemic attack, cardiac insufficiency and history of interventions), Killip classification grade and the outcomes of coronary angiography. Left ventricular ejection fraction and left ventricular end-diastolic diameter by echocardiography, and biochemical parameters including B-type natriuretic peptide (BNP), low density lipoprotein cholesterol (LDL-C), and lipoprotein A (LP(a)) were recorded.

The definition of the number of angiographic lesions was described as follows. Coronary artery and major branches were evaluated by coronary angiography. A vessel with a stenosis more than 50% was defined as a lesion vessel. When the left anterior descending coronary artery (LAD) and its branch were stenosed more than 50%, it was defined as single lesion. The definition of single lesion in the right coronary artery (RCA) or the left circumflex coronary artery (LCX) were the same as the LAD. According to the number of coronary artery lesions involved, 524 patients were divided into single vessel lesions, double vessel lesions and three vessel lesions. Patients with coronary artery disease involving the left main coronary artery were not classified separately. The severity of coronary artery disease was based on the Gensini score.

### Statistical analysis

All data were analyzed statistically using SPSS (version 23.0; IBM, Chicago, IL). Student’s *t* test was performed to compare continuous variables. Chi square test was performed to compare categorical variables. The mean of multiple groups was analyzed by one-way ANOVA. The LSD method was used for comparison among groups. If data were not normally distributed, rank sum test was used for comparison among groups. Pearson correlation analysis was used for correlation analysis. Statistical significance was set at *P* < 0.05.

## Results

The clinical data included age, gender, LVEF, LDL-C, BNP, LP(a), hypertension, hyperlipidemia, diabetes, TIA, atrial fibrillation, Killip grade and history of PCI. The correlation between CHADS_2_ and CHA_2_DS_2_-VASc and STEMI was analyzed. The data are detailed in Table 1 and Table 2, respectively. There was a significant difference in age, gender, hypertension, TIA, atrial fibrillation, Killip grade, LDL-C and BNP among the three groups in CHADS_2_ and CHA_2_DS_2_-VASc. However, there was no significant difference in LP(a), hyperlipidemia and history of PCI among the three groups by CHADS_2_ or CHA_2_DS_2_-VASc. Besides, there was a significant difference in LVEF had significant difference among three groups by CHADS_2_ but there was no difference in CHA_2_DS_2_-VASc scores.

**Table 1:** The correlation analysis of CHADS_2_ and clinical data in STEMI patients

Index	Low group (n = 205))	Middle group (n = 247)	High group (n = 72)	Z/F/χ^2^	*P*
Age	54.52 ± 9.64	63.62 ± 13.3	72.54 ± 9.89	81.026	< 0.001
Gender				25.364	< 0.001
Female	23 (4.4%)	53 (10.1%)	27 (5.2%)		
Male	182 (34.7%)	194 (37.0%)	45 (8.6%)		
LVEF	59.85 ± 9.87	57.26 ± 10.15	55.45 ± 10.43	5.398	0.005
LDL-C	3.10 ± 0.89	2.94 ± 0.94	2.67 ± 1.05	4.33	0.015
BNP	656.8 (248.1–1343.5)	1140 (485–2501)	2442 (830.1–4907)	48.07	< 0.001
LP(a)	14.81(9.1–31.7)	15.72(9–28.7)	16.04(9.95–25.85)	0.173	0.919
Hypertension	61 (11.6%)	168 (32.1%)	63 (12.0%)	103.761	< 0.001
Hyperlipidemia	170 (32.4%)	211 (40.3%)	54 (10.3%)	4.826	0.104
Diabetes	21 (4.0%)	111 (21.2%)	47 (9.0%)	97.895	< 0.001
TIA	0 (0%)	10 (1.9%)	47 (9.0%)	260.627	< 0.001
Atrial fibrillation	4 (0.8%)	14 (2.7%)	8 (1.5%)	10.487	0.005
Killip					
1	172 (32.8%)	96 (18.3%)	14 (2.7%)	172.469	< 0.001
2	32 (6.1%)	125 (23.9%)	35 (6.7%)		
≥ 3	1 (1.2%)	26 (5.0%)	23 (4.4%)		
History of PCI	9 (1.7%)	9 (1.7%)	3 (0.6%)	0.367	0.903

**Table 2:** The correlation analysis of CHA_2_DS_2_-VASc and clinical data in STEMI patients

Index	Low group (n = 184)	Middle group (n = 193)	High group (n = 147)	F/χ^2^	*P*
Age	52.62 ± 8.7	60.26 ± 10.60	72.90 ± 9.98	178.591	< 0.001
Gender				180.232	< 0.001
Female	1 (1.2%)	19 (3.6%)	83 (15.8%)		
Male	183 (34.9%)	174 (33.2%)	64 (12.2%)		
LVEF	59.58 ± 10.0	57.26 ± 10.42	56.14 ± 10.0	2.892	0.056
LDL-C	3.10 ± 0.86	2.93 ± 0.92	2.83 ± 1.00	3.038	0.048
BNP	605.6 (219.3–1214.3)	944.8 (381.2–1905)	2302.7 (1019.5–4585.5)	86.382	< 0.001
LP(a)	14.6(9.1–30)	13.8(8–27.2)	16.9(10.5–31.9)	3.621	0.167
Hypertension	55 (10.5%)	128 (24.4%)	109 (20.8%)	80.289	< 0.001
Hyperlipidemia	153 (29.2%)	163 (31.1%)	119 (22.7%)	0.978	0.645
Diabetes	21 (4.0%)	85 (16.2%)	73 (13.9%)	68.894	< 0.001
TIA	0 (0%)	9 (1.7%)	48 (9.2%)	109.581	< 0.001
Atrial fibrillation	2 (0.4%)	9 (1.7%)	15 (2.86%)	15.253	< 0.001
Killip					
1	151 (28.8%)	91 (17.4%)	40 (7.6%)	138.958	< 0.001
2	30 (5.7%)	89 (17.0%)	73 (14.0%)		
≥ 3	3 (0.6%)	13 (2.5%)	34 (6.5%)		
History of PCI	9 (1.7%)	7 (1.3%)	5 (1.0%)	0.582	0.762

According to coronary angiography, the number of coronary artery lesions was determined. The data are detailed in Table 3. In CHADS_2_ scores, there was no significant difference in the number of lesions among different groups. Meanwhile, the comparison between groups showed no significant difference. In CHA_2_DS_2_-VASc scores, there was a significant difference in the number of lesions among the different groups. The significant difference was also found between groups (the low group vs. the middle group, χ^2^ = 45.452, *P* < 0.001; the middle group vs. the high group, χ^2^ = 45.986, *P* = 0.001; the low group vs. the high group, χ^2^ = 27.315, *P* < 0.001).

**Table 3:** The comparison of CHADS_2_ and CHA_2_DS_2_-VASc with number of coronary artery lesions

Score	Group	Single lesion	Double lesions	Three lesions	χ^2^	*P*
CHADS_2_	Lower group	77	113	15		
	Middle group	87	131	29	6.128	0.190
	High group	19	42	11		
CHA_2_DS_2_-VASc	Lower group	71	102	11		
	Middle group	20	172	1	69.88	< 0.001
	High group	39	89	19		

**Fig. 1:**
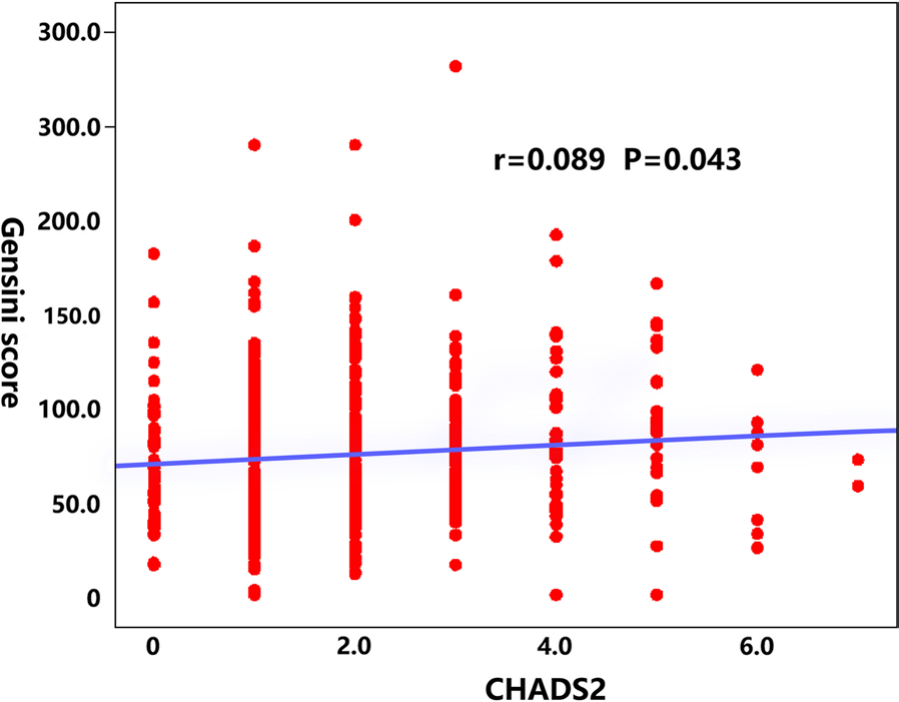
Scatter plot of correlation analysis between CHADS_2_ and Gensini scores

Gensini score can evaluate the severity of coronary artery disease. The severity of coronary artery disease increases with Gensini score. With the increase of CHADS_2_ score and CHA_2_DS_2_-VASc score, Gensini score also showed an upward trend (Table 4). There was no significant difference among all groups in both two scoring systems. When compared the subgroups, only the high group was significantly higher than the lower group in CHADS_2_ score (*P* = 0.045 < 0.05). The other comparisons between groups showed no significant difference. (the low CHADS_2_ score_,_ group vs. the middle CHADS_2_ score_,_ group, t = -0.694, *P* = 0.488, middle group vs high group, t = -1.073, *P* = 0.285; CHA_2_DS_2_-VASc score, low group vs middle group, t = -0.66, *P* = 0.51, the low CHADS_2_ score_,_ group vs. the high CHADS_2_ score_,_ group, t = -1.052, *P* = 0.294, the middle CHADS_2_ score_,_ group vs. the high CHADS_2_ score_,_ group, t = -0.401, *P* = 0.689). Pearson correlation analysis showed a positive correlation between CHADS2 score and Gensini score (r = 0.089, *P* = 0.043 < 0.05). However, there was no correlation between CHA_2_DS_2_-VASc score and Gensini score (r = 0.060, *P* = 0.174 > 0.05) (Fig. 1).

**Table 4:** Comparison of CHADS_2_/CHA_2_DS_2_-VASc with Gensini score among different groups

Gensini score	Low group	Middle group	High group	F	*P*
CHADS_2_	72.18 ± 36.81	74.77 ± 36.56	82.6 ± 39.19	2.11	0.122
CHA_2_DS_2_-VASc	72.57 ± 37.19	75.17 ± 38.76	77.22 ± 34.81	0.654	0.520

Totally 63 patients had left main coronary artery lesion. They were respectively divided into the low group, the middle group and the high group according to the evaluation criterion of CHADS_2_ and CHA_2_DS_2_-VASc score ss mentioned above. After comparison among all subgroups, the outcomes showed the prevalence of left main coronary artery disease increased significantly with the increase of CHADS_2_ score; however, there was no significant difference in CHA_2_DS_2_-VASc score (Table 5).

**Table 5:** The comparison of the cases of left main coronary artery lesion among all group in CHADS_2_ and CHA_2_DS_2_-VASc score

	Cases of left main artery lesion/total cases	χ2	*P*
CHADS_2_			
Low group	22/205		
Middle group	26/247	6.130	0.047
High group	15/72		
CHA_2_DS_2_-VASc			
Low group	20/184		
Middle group	19/193	3.671	0.160
High group	24/147		

Totally 519 patients received emergency stent implantation, and the other 5 patients underwent coronary angiography. We evaluated the correlation between coronary target vessel lesions and CHADS_2_ and CHA_2_DS_2_-VASc score. The results showed no significant difference among the different groups in both scores (Table 6).

**Table 6:** The correlation analysis between coronary target vessel lesions and CHADS_2_ /CHA_2_DS_2_-VASc score

Target vessel	Low group	Middle group	High group	χ^2^	*P*
CHADS_2_					
LAD	102 (49.8%)	115 (46.6%)	30 (41.7%)		
LCX	27 (13.2%)	30 (12.1%)	10 (13.9%)	1.840	0.765
RCA	76 (37.0%)	102 (41.3%)	32 (44.4%)		
CHA_2_DS_2_-VASc					
LAD	95 (51.6%)	83 (43.0%)	67 (45.6%)		
LCX	23 (12.5%)	21 (10.9%)	25 (17.0%)	6.861	0.143
RCA	66 (35.9%)	89 (46.1%)	55 (37.4%)		

Adverse events during hospitalization referred to death of all causes including recurrent myocardial ischemia, acute left heart failure, global heart failure, pulmonary infection, renal insufficiency, hospital stay more than 30 days, cardiac arrest, ventricular tachycardia, II ° type II and III atrioventricular block, pericardial effusion, and cardiogenic shock. Eight patients died in our study all due to cardiogenic shock. There was a significant difference in the incidence of adverse events among different groups in both CHADS_2_ and CHA_2_DS_2_-VASc score. When compared each subgroup, the high group was significantly higher than the middle group and the low group in both CHADS_2_ and CHA_2_DS_2_-VASc score (CHADS_2_, the high group vs. the middle group, χ^2^ = 13.874, *P* < 0.001; the high group vs. the low group, χ^2^ = 23.455, *P* < 0.001; CHA_2_DS_2_-VASc, the high group vs. the middle group, χ^2^ = 4.029, *P* < 0.001; the high group vs. the low group, χ^2^ = 7.414, *P* = 0.006). The middle group showed no significant difference in comparison with the low group in both scores (CHADS_2_, the low group vs. the middle group, χ^2^ = 3.349, *P* = 0.067; CHA_2_DS_2_-VASc, the low group vs. the middle group, χ^2^ = 0.917, *P* = 0.338). Kruskal–Wallis test was performed to compare the length of hospitalization due to inhomogeneity of variance (F = 29.995, *P* < 0.001). Comparing the length of hospitalization among the different groups in both CHADS_2_ and CHA_2_DS_2_-VASc score, the results showed a significant difference. When the subgroups were compared, the length of hospitalization in the high group was significantly longer than that of the middle group and the low group in both CHADS_2_ and CHA_2_DS_2_-VASc score (CHADS_2_, the high group vs. the middle group, *P* = 0.002, the high group vs. the low group, *P* < 0.001; CHA_2_DS_2_-VASc, the high group vs. the middle group, *P* < 0.001, the high group vs. the low group, *P* < 0.001). The middle group showed no significant difference in comparison with the low group in both scores (CHADS_2_, the low group vs. the middle group, *P* = 0.123; CHA_2_DS_2_-VASc, the low group vs. the middle group, *P* = 0.177) (Table 7).

**Table 7:** Comparing the adverse events/hospitalization day among different group in both CHADS_2_ and CHA_2_DS_2_-VASc score

	CHADS_2_			CHA_2_DS_2_-VASc		
	Low group	Middle group	High group	Low group	Middle group	High group
Adverse events (male/female)	0/0	3/1	4/4	1/0	3/0	3/5
χ2		55.403			22.028	
*P*		< 0.001			< 0.001	
Hospitalization day	7.28 ± 3.368	8.41 ± 4.620	12.04 ± 11.961	7.09 ± 3.318	7.76 ± 4.217	11.13 ± 9.070
H		34.019				69.474
*P*		< 0.001			< 0.001	

## Discussion

CHADS_2_ and CHA_2_DS_2_-VASc scores have been developed for risk stratification of stroke in patients with nonvalvular atrial fibrillation [9]. They are convenient scoring systems for assessing the complexity of complications. Their convenient calculation pattern makes them ideal for patients with acute coronary syndrome. Goto et al. found that CHADS_2_ score can be used not only to predict the risk of stroke in patients with atrial fibrillation, but also to predict the risk of out-of-hospital cardiovascular death in patients with established or high-risk atherosclerotic diseases [14]. Recently, many studies had confirmed that the two scores can predict the prognosis of patients with coronary heart disease, cerebral arteriosclerosis and cardiac insufficiency [15–17]. Therefore, CHADS_2_ and CHA_2_DS_2_-VASc scores may be correlated with the severity of coronary atherosclerosis.

This study is a single center retrospective study based on consecutive patients in our hospital with a definite diagnosis of "STEMI" and who had undergone emergency coronary angiography. All subjects were divided into the low group, the middle group and the high group according to CHADS_2_ and CHA_2_DS_2_-VASc scores, respectively. We analyzed several indexes, and the results showed that old age, male gender, hypertension, diabetes, cerebral infarction, TIA and atrial fibrillation were the high risk factors of AMI, which are consistent with previous studies. In the hierarchical score analysis, there was no significant difference in the prevalence of hyperlipidemia among the groups. However, the LDL-C of the low group was significantly higher than that of the middle group and the high group regardless of CHADS_2_ or CHA_2_DS_2_-VASc score. The reason for this outcome may be that the patients in the low group were younger and had fewer comorbid diseases, so were not given enough attention and appropriate treatment. On the contrary, in the middle group and the high group, the patients were older and had more complicated diseases. So, they might pay more attention to their conditions, adopting more adequate diet control and lipid lowering therapy. The increase of LDL-C was closely related to the occurrence of AMI, accounting for a higher incidence of myocardial infarction in the low group and the middle group. If the level of LDL-C was strictly controlled by diet or medication, the incidence of AMI might be reduced. Besides, LP(a) was considered a risk factor of AMI. In our study, all patients were confirmed as STEMI, this index showed no significant difference both in CHADS_2_ and CHA_2_DS_2_-VASc score.

According to the analysis of coronary angiography, the incidence of double vessel lesion was higher than that of single vessel lesion and three vessel lesion both in CHADS_2_ and CHA_2_DS_2_-VASc scores. This outcome is different from the previous study that multivessel lesion was the main coronary artery disease in STEMI. STEMI usually occurs on the basis of coronary atherosclerotic stenosis. Due to some inducements, coronary atherosclerotic plaque could rupture. After platelets gathered on the surface of the ruptured plaque and thrombi were formed. When the coronary artery lumen was blocked, it could lead to myocardial ischemia and necrosis [18, 19]. From the incidence of target vessel lesion in our study, the LAD was the most common (41.7%—51.6%), RCA was the second (35.9%—46.1%), and LCX was the lowest (10.9%—17.0%) in both CHADS_2_ and CHA_2_DS_2_-VASc score. There was no significant difference in the proportion of LAD, LCX and RCA in CHADS_2_ and CHA_2_DS_2_-VASc score. Therefore, the incidence of target vessel lesions in STMEI patients was LAD > RCA > LCX. However, this was valueless in predicting the location of target vessel lesions. As far as severe and specific left main coronary artery lesion, we found that with the increase of CHADS_2_ score, the prevalence of left main coronary artery disease increased significantly, which, however, was not seen with increase in CHA_2_DS_2_-VASc score. Gensini score is a quantitative measurement system for the coronary artery, and its calculation and application are relatively simple [20]. In recent years, many studies have evaluated the relationship between disease factors and the severity of coronary artery disease by studying the correlation between disease factors and Gensini score [21]. In our study, the results showed that with the increase of CHADS_2_ and CHA_2_DS_2_-VASc score, Gensini score showed an upward trend. Besides, there was a positive correlation between Gensini score and CHADS_2_ score, but no correlation was found between Gensini score and CHA_2_DS_2_-VASc score. This outcome might be due to the small sample size and two grading methods in this study.

In this study, we observed that there were significant differences in the incidence of adverse events and length of hospitalization among the subgroups. In these two scoring systems, the number of adverse events and length of hospitalization in the high group were significantly higher and longer than those in the middle group and the low group, but there was no significant difference between the middle group and the low group. The reason for this difference might be that the patients in the high group were older, had more complications, and had more serious vascular lesions, which led to the increase of the incidence of adverse events during hospitalization. In addition, prolonged hospitalization increased the pain of patients and hospitalization expenses. Jeong et al. [22] studied the prognosis of more than 20,000 patients with AMI who underwent PCI, showing that the higher the CHA_2_DS_2_-VASc score was, the higher the probability of in-hospital death in AMI was, which is consistent with the conclusion of our study.

However, this study still has some limitations. Firstly, this study is only a single center retrospective analysis, and the conclusions need to be further verified by large-scale multicenter prospective studies. Besides, the number of cases of left main coronary artery disease is small, which has a certain impact on the accuracy of the results. Moreover, we only selected the number of coronary artery lesions and Gensini score to evaluate the complexity of coronary artery lesions. More scoring system, such as SYNTAX and Leaman scoring method, can be used to predict lesion severity.

## Conclusion

In conclusion, the CHADS_2_ score has a certain value to predict the severity of coronary lesion and the presence of the left main coronary artery in STEMI. The CHA_2_DS_2_-VASc score has no predictive ability to do it. There was no significant predictive ability to get the number of coronary lesions and the location of the target lesions in STEMI patients. However, both scores had the predictive ability for length of hospitalization and the incidence of adverse events during hospitalization. Furthermore, these two scores are easy to obtain and calculate, rendering them more suitable for clinical application.

## Data Availability

The datasets generated and analyzed during the current study are available from the corresponding author on reasonable request.
